# Speech-language-hearing interventions in orofacial functions in individuals with Down syndrome: a scoping review

**DOI:** 10.1590/2317-1782/e20240337en

**Published:** 2026-03-27

**Authors:** Paula Rayana Batista Correia, Maria Louize Justino Freire, Julyane Feitoza Coêlho, Manuela Leitão de Vasconcelos, Andréa Rodrigues Motta, Karinna Veríssimo Meira Taveira, Giorvan Anderson dos Santos Alves

**Affiliations:** 1 Programa Associado de Pós-graduação em Fonoaudiologia, Universidade Federal da Paraíba – UFPB - João Pessoa (PB), Brasil.; 2 Departamento de Fonoaudiologia, Universidade Federal de Minas Gerais – UFMG - Belo Horizonte (MG), Brasil.; 3 Departamento de Morfologia, Centro de Biociências, Universidade Federal do Rio Grande do Norte – UFRN - Natal (RN), Brasil.; 4 Núcleo de Estudos Avançados em Revisão Sistemática e Meta-análise – NARSM, Universidade Federal do Rio Grande do Norte – UFRN - Natal (RN), Brasil.; 5 Núcleo de Inteligência Artificial em Saúde – NIAS, Universidade Federal do Rio Grande do Norte – UFRN - Natal (RN), Brasil.

**Keywords:** Myofunctional Therapy, Exercise Therapy, Rehabilitation, Stomatognathic System, Sucking, Chewing, Swallowing, Breathing, Speech, Review

## Abstract

**Purpose:**

To map the syntheses of evidence in the literature on speech-language-hearing interventions in orofacial functions and their effects on people with trisomy 21 (T21).

**Research strategies:**

A search was conducted in EMBASE, LILACS, PubMed/Medline, Scopus, Web of Science, Cochrane, and ASHA databases, also consulting grey literature.

**Selection criteria:**

The review included studies with people with T21, addressing therapies related to orofacial functions, and excluded those with other populations, studies whose intervention of interest was characterized as non-speech-language-hearing therapy or were not aimed at orofacial functions, and descriptive studies.

**Data analysis:**

Data were synthesized and described narratively, illustrated with a table and a flowchart.

**Results:**

The review included 10 studies, which involved all functions, with swallowing being the most recurrent. They were published between 2009 and 2024 and originated from various countries. Participants were of both sexes, aged 2 to 25 years. Parameters such as the number and frequency of sessions and the duration of treatment varied. The types of exercises and interventions and the evaluation methods were individualized for each study. Five studies were clinical trials and performed quantitative evaluations to verify the effectiveness of the interventions.

**Conclusion:**

The literature showed that speech-language-hearing intervention in orofacial functions in individuals with Down syndrome promotes gains and modifications in structural and functional aspects.

## INTRODUCTION

People with trisomy 21 (T21) have maxillary and mandibular growth impairments, such as maxillary hypoplasia, mandibular prognathism, dental malocclusion, narrow palate, protruded tongue and/or lodged in the floor of the mouth, and muscle hypotonia^(1)^. These aspects can affect the orofacial musculature and impair the functioning of the stomatognathic system^(1,2)^.

Orofacial structures must function properly to ensure physiological, nasal breathing. People with T21 have a greater tendency for respiratory changes such as mouth breathing and obstructive sleep apnea (OSA), influencing sleep quality, learning, memory, task performance, agitation, and the emergence of other pathologies^(3-5)^.

Moreover, tongue configurations and other oral characteristics can affect sucking, disrupting its pattern. Despite the little scientific evidence, studies report that babies with T21 may have impaired breastfeeding effectiveness, with delays in achieving intraoral vacuum and adequate sucking duration^(6,7)^.

Individuals with T21 often have masticatory dysfunction in feeding, potentially impacting nutrition and quality of life, although few studies have investigated this function^(8,9)^. Swallowing can also be affected in T21, with a more complex and less coordinated performance due to lower muscle tone and efficiency. Thus, children with T21 may have higher rates of food selectivity, coughing during swallowing, and abnormal swallowing function^(10,11)^.

Furthermore, studies indicate that people with T21 may tend to develop musculoskeletal speech disorders. These are the most common types, in which speech alterations originate from structural bone and muscle changes, resulting in phonatory, articulation, resonance, and prosody disorders, affecting their speech intelligibility^(12-14)^.

Therefore, speech-language-hearing (SLH) therapy can offer specific exercises and techniques that help improve the performance and functioning of oral structures and skills, improving the development of the stomatognathic system in people with T21, allowing for better quality in broad aspects of life^(15)^. This requires scientific support to underpin the use of strategies for safety and efficiency in the intervention.

This research aimed to conduct a literature review, considering the lack of review studies addressing SLH interventions in orofacial functions in individuals with T21. This will help to elucidate the main available evidence in the field and highlight existing gaps, indicating areas of interest for future research. Thus, this study aimed to map the literature to synthesize evidence on SLH interventions in orofacial functions and their effects on individuals with T21.

## METHODS

This scoping review followed the guidelines of the Joanna Briggs Institute (JBI) manual^(16)^. Therefore, both the protocol and this review were prepared using the checklist of the Preferred Reporting Items for Systematic Reviews and Meta-Analyses – Extension for Scoping Reviews (PRISMA-ScR)^(17)^. The protocol for this study was registered on the Open Science Framework^(18)^.

### Eligibility criteria

The acronym PCC (Participants, Concept, Context) was used to assess the eligibility criteria, as follows: Participants: people with T21; Concept: functions of the stomatognathic system, such as sucking, breathing, chewing, swallowing, and speech (including residual speech errors); Context: therapy. The following research question was formulated based on these criteria: “What scientific evidence is available in the literature on SLH interventions in the functions of the stomatognathic system in people with T21?”.

The review included studies with people with T21, addressing SLH therapies related to sucking, breathing, chewing, swallowing, and speech (including residual speech errors). The types of studies included were clinical trials, case series, and case studies.

Studies involving other populations were excluded from this review, as well as studies that presented SLH interventions not directed at orofacial functions, whose intervention of interest was characterized as non-SLH therapy (i.e., therapeutic approaches such as acupuncture, medication use, or surgery), descriptive studies (cross-sectional, cohort, and case-control studies), and secondary studies (e.g., abstracts, systematic, narrative, and integrative reviews, meta-analyses, letters to the editor, guidelines, clinical practice guidelines, and expert opinions). There were no exclusion criteria based on ethnicity, sex, age, language, or year of publication of the study.

### Sources of information and search strategies

The search strategy was applied to electronic searches of scientific publications indexed in the following databases: ASHA, Embase, PubMed/Medline, LILACS, Scopus, Cochrane, and Web of Science. Grey literature was also used as a source of information through Google Scholar and ProQuest. In addition, the references of the included studies were manually searched. The Boolean operators OR and AND were used to combine the descriptors (therapeutics, mastication, deglutition, speech, respiration, sucking behavior, Down syndrome) and their correlates. These terms were selected from the Health Sciences Descriptors (DeCS/MeSH) and EMTREE. All searches were performed on August 11, 2023, and updated on February 5, 2025 (Appendix A).

### Selection

The sources of evidence were selected in three stages. The Rayyan^®^ website (Rayyan QCRI)^(19)^ analyzed the inclusion and exclusion of articles, and the Endnote^®^ reference manager (EndNote^®^ X7 Thomson Reuters, Philadelphia, PA) removed duplicates.

Two independent reviewers were calibrated on the Rayyan website to assess their level of agreement. They applied the eligibility criteria to determine the inclusion and exclusion of studies and analyzed the titles and abstracts of 100 articles selected from the literature, using the research question.

Good agreement was found for titles and abstracts, with a kappa agreement coefficient > 0.8. Then they screened the remaining studies from the first phase.

In the second phase, they read the full texts and applied the eligibility criteria. The two reviewers selected studies independently, and any disagreements were resolved by a third reviewer. The latter, with greater clinical experience, was present at all stages of data collection to analyze and resolve disagreements, select relevant research on the topic, and guide practice based on scientific knowledge.

### Data extraction and analysis

The data were extracted according to a specific protocol developed by the authors. They analyzed publication data, methodological characteristics (e.g., sample size, measurement of variables, and method of analysis), description of the population, processes of orofacial function interventions (including the number of sessions, frequency and format of the intervention, and the description of the exercises), evaluation (including instruments used and results), temporal parameters, the identification of results (positive, negative, or neutral), and the effectiveness of the program or intervention.

The analyses and presentation of the data were synthesized and described in a narrative, illustrated with a table and a flowchart.

## RESULTS

### Selection of sources of evidence

**Figure 1:**
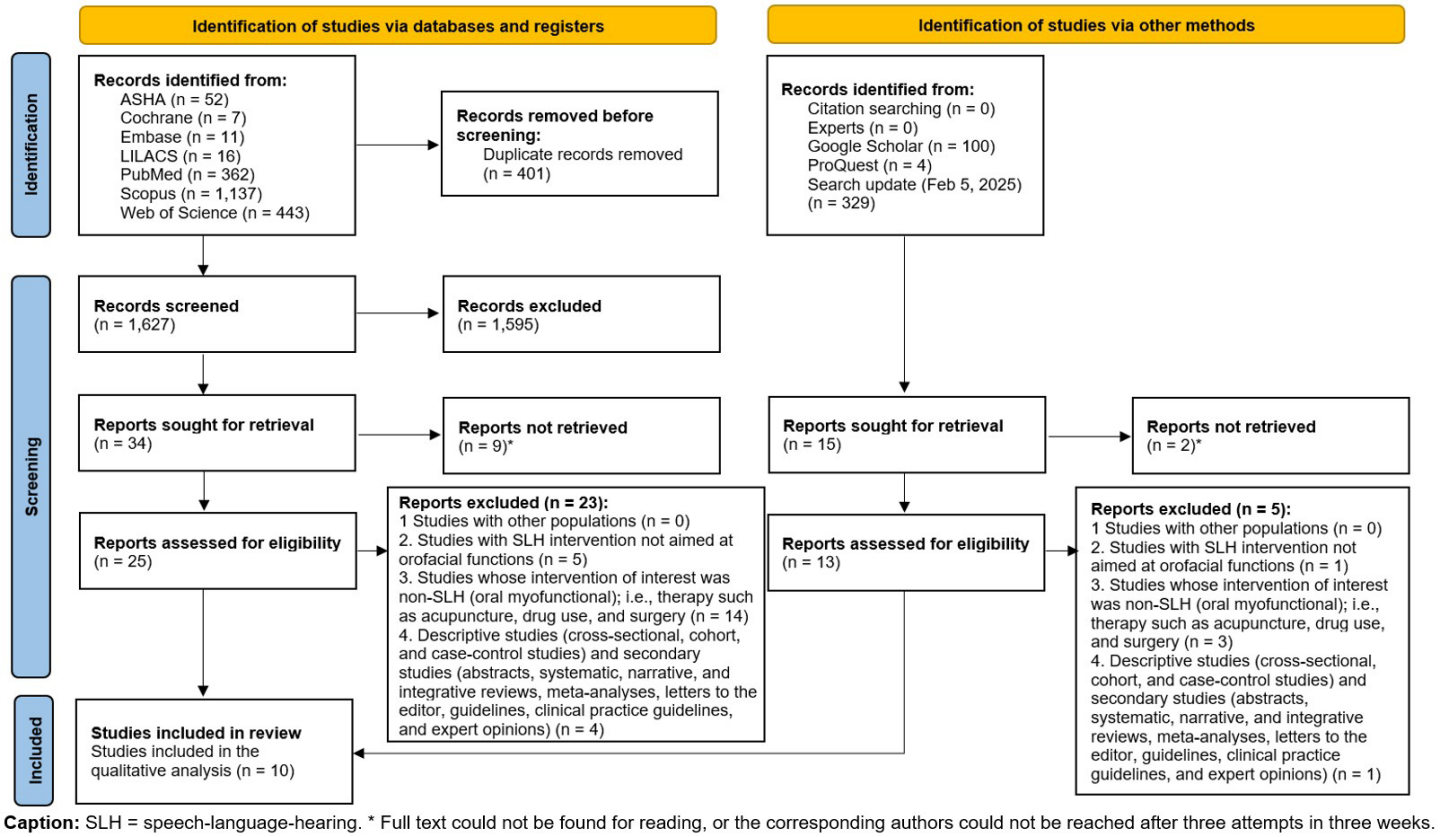
Flowchart for selecting sources of evidence.

The search identified a total of 2,028 articles in the databases and 433 studies in grey literature repositories, as illustrated in Figure 1. After removing duplicates, 2,429 citations were selected (phase 1). The analysis of titles and abstracts excluded 2,013 studies, leaving 49 articles for retrieval and full-text evaluation for eligibility (phase 2). However, 11 studies were not retrieved in full, and 28 studies were excluded after evaluating the full texts. Hence, at the end of the process, 10 studies were considered eligible for this scoping review.

### Characteristics of the studies

Chart 1 presents the configurations of the included studies, divided by authors, year, country, study type, participant characteristics (sample and age), training programs (number of sessions, session frequency, type of exercise), SLH interventions, assessment methods, and results.

**Chart 1:** Characteristics of the studies included in the review

**Author, Year, Country**		**Study type**	**Characteristics of participants**					**Training program**							**Evaluation of results**	**Results**
			**Sample**			**Age**		**Number of sessions**		**Frequency and duration of sessions**		**SLH interventions (orofacial functions)**	**Description of exercises**			
Anichini et al.^(20)^, 2013. Italy		Case study	n = 7 (3 with BWS, 2 with DS, and 2 with other diagnoses			4-14 years		DS1: NR DS2: Continuous		DS1: NR DS2: 3 times a week Duration NR		Macroglossia and swallowing	Individualized intervention: DS1: Exercises to correct macroglossia (stimulation of orofacial praxis) and to improve language DS2: SLH exercises		SLH assessment: Recording vocal parameters, phonetics, articulation, gestural and oral communication, assessment of graphic skills, writing, reading, rhythmic and perceptual levels, global and fine motor skills, behavioral scheme. - Great attention was paid to performing oral-lingual movements, observation of the perioral muscles, tongue, lips, and jaw, and the assessment of breathing, swallowing, and chewing skills. - Physical, laboratory, and instrumental examinations	DS1: Improved concentration, behavior, attention, and phonatory skills, reduced tongue protrusion, strengthened muscle tone DS2: Good responses in macroglossia, atypical swallowing and language
Putri et al.^(21)^, 2021. Indonesia		Randomized clinical trial	n = 41 Control group: 21 Intervention group: 20			3-9 years		21 days/sessions		1-hour session, 3 times a day		Sucking	Sucking exercise – sucking plain water using straight and circular straws		Clinical Examination and Mouth Rinsing Test Function (MRT-F) Scale Mouth rinsing test: a) Transferring 10 ml of water from the bowl to the mouth b) Closing the lips and holding the water in the mouth c) Moving the two cheeks symmetrically and moving the cheeks alternately e) Disposing of the water in the sink	The 1^st^-day MRT-F scores were 1 (30%), 2 (35%), 3 (20%), and 4 (15%) in the circular straw group, and 1 (38.2%), 2 (19%), 3 (42.9%), and 4 (0%) in the straight straw group. At the end of the 3^rd^ week, the scores were 1 (25%), 2 (25%), 3 (15%), 4 (20%), and 5 (15%), while in the straight straw group they were 1 (33%), 2 (19%), 3 (28.6%), 4 (14.3%), and 5 (4.8%). - MRT-F scores differed significantly before and after the exercises. Mouth rinsing capacity in children with DS improved after performing water suction exercises in both groups. However, the exercises with straight and circular straws did not differ significantly.
Saccomanno et al.^(22)^, 2018. Rome		Pilot intervention study, not randomized	n = 10			9-18 years		20 weeks		Each objective: a series of 5 exercises. At home: repeat each exercise at least 3 times a day, totaling 30 minutes of MT per day		1- Nose functioning 2- Nasal breathing 3- Tongue position 4- Lip sealing 5- Buccinator and masseter muscles 6- Soft palate 7- Swallowing	1^st^ exercise: Nasal hygiene: techniques for washing nasal passages and learning how to blow the nose correctly 2^nd^ exercise: Keeping the mouth closed and inhaling and exhaling, keeping a tongue depressor between the lips. 3^rd^ exercise: Stimulating first the anterior portion of the tongue, then the lateral portion, and finally the posterior portion to adapt the position of the tongue 4^th^ exercise: Exercises to restore lip sealing 5^th^ exercise: Chewing, jaw stability, and sustained inflated cheek exercises 6^th^ exercise: Gargling and energetic pronunciation of vowel phonemes and single vowels 7^th^ exercise: Techniques to repattern tongue movement to achieve adequate swallowing		Oral myofunctional, cognitive, and receptive and expressive language assessment, and a tongue-mouth-face praxis exam	All had good ability to clear and relieve nasal passages by blowing their nose or performing nasal irrigation. All had habitual nasal breathing ability. Four participants achieved a resting tongue posture on the palate. All improved in lip seal during feeding but remained inconsistent at rest. All improved masseter muscle tone and chewing ability and increased palatal mobility. All improved swallowing and reduced sialorrhea, rhinorrhea, or angular cheilitis.
Von Lukowicz et al.^(23)^, 2019. Germany		Non-randomized experimental study	n = 42			2 – 11 years		1 week = 7 sessions		Three 45-minute sessions a day		Breathing – obstructive sleep apnea	Padovan Method: physical exercises to strengthen general muscle tone and improve posture, followed by oral exercises to control air flow, lip activity, tongue movements to increase strength, activation of the buccinator and masseter muscles, and proprioception through chewing exercises. Oral exercises to strengthen the orofacial complex and improve intake, articulation, and development of the face and jaws.		Nighttime polygraphy	MOAHI (obstructive apnea/hypopnea index), DI3 (desaturation index), and SpO2nadir (lowest oxygen saturation value) did not differ before and after MT. Only DI90 (desaturation index) decreased. The median MOAHI decreased more in children without prior MT (from 6.1 to 1.5 vs. from 2.7 to 2.2).
Cleland et al.^(24)^, 2009. Scotland		Randomized clinical trial	n = 6			10 – 18 years		24 individualized therapy sessions		Twice a week for 12 weeks 1 hour at the clinic. At home: 10 to 15 minutes, 5 days a week		Speech	Starting with CV or VC target phoneme, progressing to CV and VC with different vowels, words (construction complexity), and then sentences and conversation in speech Participants were initially encouraged to copy the target pattern using the EPG for visual feedback, progressing to productions without visual feedback. Individualized program		Language assessment: (BPVS-II, Dunn et al.^(25)^) CELF-P, Wiig et al.^(26)^). Cognitive ability: (Wechsler Primary and Preschool Scale of Intelligence) and (WPPSI-IIIUK, Wechsler^(27)^). Oral-motor Function: (Robbins and Klee^(28)^ Clinical Assessment) Pre and post: - EPG measures. - Phonology (DEAP, Dodd et al.^(29)^)	PCC increased significantly from 63.50% to 73.67% (t(5) = 3.634, p = 0.015), with an average increase of 10.17% per child. The number of words correctly identified by listeners increased rapidly but not significantly post-intervention, from 63.5% to 66.06% (t(5) = 0.616, p = 0.565) on the CSIM. As a group, positive changes were evident in five of the six participants; participant 2 had no change in EPG patterns, but a shift to a more appropriate airflow. Perceptually, all children except participant 2 increased the number of perceptually correct productions. The overall increase was significant (t(5) = 3.286. All participants improved the percentage of correct consonants, and most participants produced visibly different EPG patterns after 24 sessions of EPG visual feedback therapy.
Alves Pinheiro et al.^(2)^, 2018. Brazil		Non-randomized intervention study	n = 16			9-25 years		8 sessions		Weekly Duration NR		Chewing	Chewing training (with Bono sandwich cookies), always with commands to induce volunteers to chew bilaterally and alternately, and, when possible, with lip sealing. Electrostimulation		Orofacial myofunctional assessment using the Orofacial Myofunctional Evaluation Protocol with Scores (OMES)	Significant change in lip posture, maintaining sealed lips. Significant change in cheek appearance, reducing flabbiness. Difference before and after intervention in tongue lateralization, both to the right and left sides. Improved breathing. Improved lip behavior during swallowing. In chewing, the bite and grinding differed significantly between before and after intervention, which aimed for biting with the incisors and grinding with a normal bilateral alternating pattern.
Wood et al.^(30)^, 2018. Scotland		Randomized clinical trial	n = 27 Group 1 therapy: EPG directly; VBF Group 2 therapy: conventional therapy informed by pre-therapy EPG assessment Group 3 therapy: control group, usual treatment			8-18 years		For 12 weeks 24 sessions		Twice a week, with 1-hour sessions		Speech	Speech therapy associated with EPG: Therapy followed a basic articulatory hierarchy, starting with the target phoneme in CV or VC structure, using a facilitating vowel, and progressing to other vowels and CVC structures, continuing to more complex word structures. Where progress indicated such a possibility, SLT moved to the phrase and sentence level		Cognitive ability: Scale (WPPSI-IIIUK; Wechsler^(27)^) Receptive vocabulary: (BPVS-II; Dunn et al.^(25)^) Receptive and expressive language: (CELFP-UK, Wiig et al.^(26)^): Oral-motor function: Clinical assessment of oropharyngeal motor development (Robbins and Klee^(28)^) Speech production: Phonology subtest, Assessment of Articulation and Phonology (DEAP; Dodd et al.^(29)^): Intelligibility: Child Speech Intelligibility Measure (CSIM; Wilcox and Morris^(31)^)	According to the DEAP PCC difference scores pre- and post-therapy, there was no statistically significant difference between the intervention groups at this time: ÿ2 (2) = 1.830, p = 0.00. 401
Homer and Carbajal^(32)^, 2015. United States		Case study	N = 1			5 years		NR		Daily Duration NR		Swallowing	Swallowing and feeding plan that addresses positioning, equipment, feeding status, diet, food preparation, and feeding plan techniques and precautions. Desensitization through oral motor stimulation using a Nuk brush before meals, and incorporating exercises to improve tongue lateralization, lip closure, and general oral motor skills for eating. Techniques to reduce tongue protrusion Trying different cups and reducing the use of straws. Gradually increasing the variety, texture, and amount of foods and liquids consumed during the school day, adding one or two new foods at a time. Techniques to promote independent feeding, encouraging finger and spoon feeding by placing them on the molars and giving verbal instructions to chew. Self-feeding techniques		NR	It helped increase their tolerance to denser textures and stimulate chewing.
Junqueira et al.^(33)^, 2023. Brazil	Case study			N = 1	3 years and 2 months		6 months		Once a week Duration NR		Chewing			Vestibular (swing), tactile, and proprioceptive stimuli (strength activities, vibration, and massages Tactile-thermal-gustatory stimulation, using water flavored with citrus fruit and textured teethers. Food offered throughout the session, encouraging the placement of food in the molar region, favoring tongue lateralization and, consequently, chewing. The same food was offered in various forms: whole, cut, crumbled, with a finger, with a fork, with a textured spoon, etc., varying the sensory-motor-oral pattern.	SLH assessment conducted in person, using foods the child expressed interest in and with the aid of familiar utensils (spoon and teething rings)	The menu was expanded to include all types of food, an improvement in perception and intraoral motor skills, acceptance of spoons and forks of different sizes, and ways of presenting food (whole, cut and crumbled - offered with cutlery or with the hands), autonomy and pleasure in meals.
Franceschetti et al.^(34)^, 2024. Italy	Pilot intervention study, not randomized.			N = 20	1-18 years		15 sessions		3 sessions per day, 5 consecutive days Duration NR		Chewing			Global Intensive Feeding Therapy (GIFT) Program	Pediatric Screening – Priority Evaluation Dysphagia (PS-PED) Karaduman Chewing Performance Scale (KCPS) International Dysphagia Diet Standardisation Initiative (IDDSI)	There were statistically significant improvements in chewing performance, measured by the KCPS (p < 0.01), and in texture acceptance and modification, measured by the post-intervention IDDSI (p < 0.01). A large effect size was found in KCPS and IDDSI (Kendall's W value > 0.8).

Caption: SLH: speech-language-hearing; BWS: Beckwith-Wiedemann syndrome; DS: Down syndrome; DS1: Down syndrome patient number 1; DS2: Down syndrome patient number 2; NR: not reported; MT: myofunctional therapy; EPG: electropalatography; VBF: Visual biofeedback; PCC: percentage of consonants correct; MRT-F: Mouth Rinsing Test Function; MOAHI: Mixed Obstructive Apnea/Hypopnea Index; DI90: desaturation index; CV: consonant vowel; VC: vowel consonant; CVC: Consonant Vowel Consonant

The included studies were published between 2009 and 2024 in different journals and conducted in various countries, such as Brazil^(2,33)^, Italy^(20,22,34)^, Indonesia^(21)^, Germany^(23)^, Scotland^(24,30)^, and the United States^(32)^.

All participants had a diagnosis of T21, encompassed both sexes, and were aged 2 to 25 years. One study addressed swallowing and other structural aspects of the stomatognathic system^(20)^, another focused exclusively on sucking^(21)^, one covered breathing, chewing, and swallowing^(22)^, while another focused on breathing^(23)^. Two analyzed speech^(24,30)^, three addressed chewing^(2,33,34)^, and one addressed swallowing only^(32)^.

The number of sessions varied significantly, ranging from seven to continuous treatment. One study involved only one therapeutic session, which was complemented by training family members to carry out the intervention at home^(21)^. The frequency of treatment also differed considerably between studies. The duration of treatments ranged from 1 to 12 weeks, although some studies did not describe this information in detail^(20,32)^. The types of exercises and interventions were also specific to each study; some were individualized, and others followed the same pattern for all participants. The assessment methods used specific protocols and techniques for each area of ​​interest.

Three studies were randomized clinical trials^(21,24,30)^, four were non-randomized clinical trials^(2,22,23,34)^, and three were case studies^(20,32,33)^. Five studies performed qualitative assessments to verify the effectiveness of the interventions^(20,22,23,32,33)^, while five studies carried out quantitative analyses^(2,21,24,30,34)^.

Some studies suggested increasing the sample size, considering cognitive ability, the severity of structural and functional changes, access to other treatments, the intervention time, and the intervention protocol to achieve stable results over time^(21,23,30,34)^.

### Individual study results

#### Suction

The review found only one interventional study on sucking function in people with T21^(21)^. It performed exercises to suck pure water using straight and circular straws. The data were evaluated quantitatively, demonstrating improved mouth rinsing skills in both groups after the exercises. However, the results of exercises with straight and circular straws were not significantly different, suggesting the need for further investigation with different methods^(21)^.

#### Breathing

This research identified one study^(23)^ on OSA, which involved exercise-based interventions with the Padovan Method. After a quantitative analysis, it was concluded that intense myofunctional training, performed for 1 week and evaluated in children with T21, had only a marginal effect on OSA. The study suggests that a longer follow-up or longer intervention could produce more significant effects^(23)^.

#### Chewing

Three studies approached chewing. In one of them, the intervention consisted of chewing training associated with electrostimulation^(2)^. The results, analyzed statistically, showed that electrostimulation combined with chewing training had positive effects on the masseter muscles, resulting in functional gains in chewing, breathing, and swallowing in people with T21^(2)^.

The second^(33)^ presented vestibular, tactile, and proprioceptive stimuli, strength activities, vibration, massage, and masticatory training. The results, analyzed qualitatively, demonstrated an improvement in intraoral motor skills, autonomy, and pleasure in meals^(33)^.

The third study^(23)^ based the intervention on an intensive feeding therapy program (GIFT) with techniques that provided statistically significant results in masticatory performance^(23)^.

#### Swallowing

One of the studies on swallowing^(20)^ individualized the interventions per patient, applying macroglossia correction exercises, orofacial praxis stimuli, language-focused exercises, and SLH exercises. The results, expressed qualitatively, indicated that all the children in the study had a good response and adherence, with notable improvements, demonstrating the importance of early, consistent, and intensive treatment^(20)^.

Another study focused on swallowing^(32)^, with an intervention plan for swallowing and feeding. The results, also expressed qualitatively, concluded that the intervention helped to increase tolerance to denser food textures and to stimulate chewing^(32)^.

#### Speech

One of the studies on speech function^(24)^ individualized interventions. It followed a hierarchical pattern, starting with target phonemes and progressing to variations, words, phrases, and conversation in speech. It also used electropalatography (EPG) for visual feedback, progressing to productions without this feedback. The results, assessed quantitatively, showed qualitative and quantifiable differences in EPG patterns and improvements in the percentage of correct consonants, indicating that EPG is a promising approach to identify and improve articulatory patterns in people with T21^(24)^.

Another study on speech^(30)^ likewise applied EPG therapy to participants. Statistical analysis showed that the intervention effectively improved speech production in many participants. However, the authors stressed the need for further investigation with a larger sample^(30)^.

#### Stomatognathic functions and structural aspects

A study addressed an intervention protocol for T21, with exercises for nasal functioning, nasal breathing, tongue position, lip seal, strengthening and mobility of buccinator and masseter muscles, soft palate, and swallowing. The results were described qualitatively, indicating improvements in several aspects worked on, such as the adequate restoration of orofacial physiological kinetics and oral functions (e.g., swallowing, breathing, and articulation)^(22)^.

## DISCUSSION

SLH interventions focused on orofacial functions in individuals with T21 have demonstrated the potential to promote relevant clinical improvements in aspects such as sucking, breathing, chewing, swallowing, and speech. These results reinforce the importance of SLH therapy in promoting the functioning of the stomatognathic system in this population.

The included studies were conducted between 2009 and 2024^(2,20-24,30,32-34)^, but only three of them were published in the last 5 years^(21,33,34)^. This highlights the scarcity of research in the area and emphasizes the imminent need for expansion.

The study participants were mainly children and adolescents with T21^(20-24,30,33,34)^, while only one included adults with T21^(2)^. These findings demonstrate a greater tendency for studies focused on the early periods of life, which may be due to early SLH stimulation prioritizing the first years of life^(15,35)^.

Most studies had few participants with T21, ranging from 1 to 20^(2,20-22,24,30,32-34)^; only one had a larger sample, with 42 individuals^(23)^. These aspects may be related to difficulties in recruiting participants and having them adhere to the therapeutic process, common challenges in scientific research with this population.

Other relevant methodological considerations, observed in most studies, include the lack of a control group^(2,20,22,23,32,34)^ and the use of qualitative analyses to evaluate the effects of the interventions^(20,22,23,32,33)^. These factors make it difficult to understand which differential aspects should be implemented in interventions aimed at this population, due to the lack of comparative parameters obtained from individuals without T21 undergoing the same intervention.

Regarding the intervention on sucking in T21^(21)^, the exercises included sucking water through a straw, three times a day, for 1 hour. However, the authors suggested methodological changes to obtain more consistent results^(23)^. Difficulty sucking in babies with T21 is a relevant factor, since it can compromise breastfeeding due to delays in obtaining intraoral vacuum and in the duration of sucking^(6,7,36)^.

Breathing results with the Padovan Method were not satisfactory in OSA, possibly due to the short follow-up and the small sample size^(23)^. This method consists of a sequence of motor, respiratory, and oral reflex-vegetative stimuli exercises, with a potential impact on respiratory function^(37)^.

In chewing, only one study used masticatory training associated with electrostimulation, demonstrating positive effects in the population with T21. Moreover, specific intervention in the masticatory musculature improved other stomatognathic functions^(2)^, while the other two studies used conventional therapy in the area, without the aid of technological resources, also presenting good structural and functional results^(33,34)^.

Swallowing findings were presented qualitatively, revealing improved positioning and tonicity of orofacial structures and increased tolerance to firmer textures^(20,32)^. This information reinforces the need for controlled studies to evaluate the long-term effectiveness of these interventions.

Concerning speech, one study found improved percentage of correct consonants and differentiated electropalatogram patterns after intervention^(24)^. Another study compared conventional therapy with therapy mediated by EPG visual feedback, with no statistically significant differences between the groups, although there was a gain in production accuracy^(30)^.

One study used intervention in more than one function, achieving functional improvements in swallowing, breathing, and speech^(22)^. The stimulation likely provided changes in neuromuscular aspects, enhancing more than one function of the stomatognathic system.

The findings analyzed indicate that SLH therapy focused on orofacial functions in T21 has beneficial effects, including improvements in functions not directly worked on^(2,21,32)^. This result corroborates the proposal of orofacial myofunctional therapy, which seeks to recover the functioning of the stomatognathic system through techniques that facilitate neuromuscular action^(38)^.

Conventional approaches were predominant among the included studies^(20-23,32-34)^, although some used technological resources to enhance the effects of the intervention^(2,24,30)^. The scarcity of studies in the area highlights the need for further investigation to obtain greater scientific support.

The difficulty in accessing full texts and the lack of detail in some intervention methods stand out among the limitations identified, making data extraction difficult. Moreover, the lack of standardization in assessment and intervention protocols compromises the comparability of results. The small sample sizes also limit the performance of more precise quantitative analyses, making it difficult to obtain more robust evidence.

Lastly, most studies were not randomized controlled clinical trials, considered the gold standard for health research^(39)^. This reinforces the need for more methodologically rigorous research to improve the evidence in the area.

The limited number of studies available highlights the urgency of expanding investigations, with an emphasis on larger samples, control groups, robust quantitative analyses, and longitudinal follow-up. Future studies should explore the various orofacial functions, especially swallowing, which only two case studies investigated. Standardizing specific therapeutic approaches for each function is also recommended, considering different age groups and proposing strategies based on the best available evidence in oral motor therapy.

## CONCLUSION

Sucking test results based on a mouthwash test demonstrated evidence of improvements in function. As for breathing function, the intervention had only a marginal effect on individuals with OSA. Furthermore, interventions targeting breathing and swallowing functions improved not only these oral functions but also speech articulation.

The findings related to chewing demonstrated that electrostimulation associated with masticatory training provided functional gains in chewing, breathing, and swallowing functions. Likewise, conventional therapy improved chewing performance and intraoral motor skills, promoting autonomy and pleasure in meals. Studies focused on swallowing demonstrated improved function, increased tolerance to denser textures, and stimulated chewing. Studies involving speech identified changes in EPG patterns and improvements in speech production after the intervention.

This study makes important contributions to the clinical context, as it highlights the available evidence on interventions targeting orofacial functions, guiding professionals in developing more effective therapeutic strategies, especially those working with interventions for individuals with T21, through techniques that enhance the intervention.

## Appendix A Search strategies

**Table:**

**Databases**	**Search** (on August 11, 2023, updated on February 5, 2025)
**ASHA**	("Down Syndrome" OR "Mongolism" OR "Trisomy G" OR "Down's Syndrome" OR "Downs Syndrome" OR "Trisomy 21") AND ("myofunctional therapy" OR "myofunctional therapies" OR "orofacial myotherapy" OR "oral myotherapy" OR "orofacial myology" OR "oral exercise" OR "exercise therapy" OR "remedial exercise" OR "rehabilitation exercise" OR "habilitation" OR "therapeutics" OR "therapy" OR "Therapeutic" OR "Therapies" OR "Treatment" OR "Treatments" OR "intervention" OR "interventions") AND ("Mastication" OR "chewing" OR "chewing ability" OR "chewing performance" OR "masticatory ability" OR "masticatory function" OR "masticatory disability" OR "masticatory dysfunction" OR "chewing function" OR "chewing discomfort" OR "chewing disorder" OR "chewing problems" OR "Sucking Behavior" OR "Sucking Behaviors" OR "sucking")
**Cochrane**	("Down Syndrome" OR "Mongolism" OR "Trisomy G" OR "Down's Syndrome" OR "Downs Syndrome" OR "Trisomy 21") in Title Abstract Keyword AND ("myofunctional therapy" OR "myofunctional therapies" OR "orofacial myotherapy" OR "oral myotherapy" OR "orofacial myology" OR "oral exercise" OR "exercise therapy" OR "remedial exercise" OR "rehabilitation exercise" OR "habilitation" OR "therapeutics" OR "therapy" OR "Therapeutic" OR "Therapies" OR "Treatment" OR "Treatments" OR "intervention" OR "interventions") in Title Abstract Keyword AND ("Mastication" OR "chewing" OR "chewing ability" OR "chewing performance" OR "masticatory ability" OR "masticatory function" OR "masticatory disability" OR "masticatory dysfunction" OR "chewing function" OR "chewing discomfort" OR "chewing disorder" OR "chewing problems" OR "Sucking Behavior" OR "Sucking Behaviors" OR "sucking") in Title Abstract Keyword
**Embase**	#1. 'down syndrome'/exp OR 'down syndrome' OR 'mongolism'/exp OR 'mongolism' OR 'trisomy g'/exp OR 'trisomy g' OR 'downs syndrome'/exp OR 'downs syndrome' OR 'trisomy 21'/exp OR 'trisomy 21'
	#2. 'myofunctional therapy' OR 'myofunctional therapies' OR 'orofacial myotherapy' OR 'oral myotherapy' OR 'orofacial myology' OR 'oral exercise' OR 'promotion program' OR 'long-term care prevention programs' OR 'resistance training' OR 'strength training' OR 'exercise program' OR 'exercise therapy' OR 'remedial exercise'
	#3. 'stomatognathic system' OR 'masticatory system' OR 'mastication' OR 'chewing' OR 'deglutition' OR 'deglutitions' OR 'swallowing' OR 'swallowings' OR 'speech' OR 'respiration' OR 'breathing' OR 'sucking behavior' OR 'sucking behaviors' OR 'sucking'
	#4. #1 AND #2 AND #3
**LILACS**	("Down Syndrome" OR "Mongolism" OR "Trisomy G" OR "Down's Syndrome" OR "Downs Syndrome" OR "Trisomy 21") AND ("Myofunctional Therapy" OR "myofunctional therapies" OR "orofacial myotherapy" OR "oral myotherapy" OR "orofacial myology" OR "oral exercise" OR "exercise therapy" OR "remedial exercise" OR "rehabilitation exercise" OR "rehabilitation" OR "habilitation" OR "terapia miofuncional" OR "treinamento de força" OR "terapia por exercício" OR "reabilitação" OR "habilitação" OR "terapia miofuncional" OR "entrenamiento de fuerza" OR "terapia por ejercicio" OR "rehabilitación" OR "habilitacion") AND ("Stomatognathic System" OR "Masticatory System" OR "Mastication" OR "Chewing" OR "Deglutition" OR "Deglutitions" OR "Swallowing" OR "Speech" OR "Respiration" OR "Breathing" OR "Sucking Behavior" OR "Sucking Behaviors" OR "sucking" OR "sistema estomatognático" OR "sistema mastigatório" OR "mastigação" OR "deglutição" OR "fala" OR "respiração" OR "sistema estomatognático" OR "sistema masticatorio" OR "masticación" OR "deglución" OR "habla" OR "respiración" OR "Comportamento de Sucção" OR "Conducta en la Lactancia")
**PubMed/Medline**	#1. ("Down Syndrome"[Mesh] OR "Down Syndrome" OR "Mongolism" OR "Trisomy G" OR "Down's Syndrome" OR "Downs Syndrome" OR "Trisomy 21")
	#2. ("Myofunctional Therapy"[MeSH] OR "myofunctional therapy" OR "myofunctional therapies" OR "orofacial myotherapy" OR "oral myotherapy" OR "orofacial myology" OR "oral exercise" OR "exercise therapy"[MeSH] OR "remedial exercise" OR "rehabilitation exercise" OR "rehabilitation"[MeSH] OR "habilitation" OR "therapeutics"[MesH] OR "therapeutics" OR "therapy" OR "Therapeutic" OR "Therapies" OR "Treatment" OR "Treatments" OR "intervention" OR "interventions")
	#3. ("Stomatognathic System"[MeSH] OR "Masticatory System" OR "Mastication"[MesH] OR "mastication" OR "Chewing" OR "Deglutition"[Mesh] OR "Deglutitions" OR "Swallowing" OR "Swallowings" OR "Speech"[Mesh] OR "Respiration"[Mesh] OR "Breathing" OR "Sucking Behavior"[Mesh] OR "Sucking Behavior" OR "Sucking Behaviors" OR "sucking")
	#4. #1 AND #2 AND #3
**Scopus**	("Down Syndrome" OR "Mongolism" OR "Trisomy G" OR "Down's Syndrome" OR "Downs Syndrome" OR "Trisomy 21") AND TITLE-ABS-KEY ("myofunctional therapy" OR "myofunctional therapies" OR "orofacial myotherapy" OR "oral myotherapy" OR "orofacial myology" OR "oral exercise" OR "exercise therapy" OR "remedial exercise" OR "rehabilitation exercise" OR "habilitation" OR "therapeutics" OR "therapy" OR "Therapeutic" OR "Therapies" OR "Treatment" OR "Treatments" OR "intervention" OR "interventions") AND TITLE-ABS-KEY ( "Stomatognathic System" OR "Masticatory System" OR "mastication" OR "Chewing" OR "Deglutition" OR "Deglutitions" OR "Swallowing" OR "Swallowings" OR "Speech" OR "Respiration" OR "Breathing" OR "Sucking Behavior" OR "Sucking Behaviors" OR "sucking")
**Web of Science**	("Down Syndrome" OR "Mongolism" OR "Trisomy G" OR "Down's Syndrome" OR "Downs Syndrome" OR "Trisomy 21") (Tópico) AND ("myofunctional therapy" OR "myofunctional therapies" OR "orofacial myotherapy" OR "oral myotherapy" OR "orofacial myology" OR "oral exercise" OR "exercise therapy" OR "remedial exercise" OR "rehabilitation exercise" OR "habilitation" OR "therapeutics" OR "therapy" OR "Therapeutic" OR "Therapies" OR "Treatment" OR "Treatments" OR "intervention" OR "interventions") (Tópico) AND ("Stomatognathic System" OR "Masticatory System" OR "mastication" OR "Chewing" OR "Deglutition" OR "deglutition" OR "Swallowing" OR "swallowing" OR "Speech" OR "Respiration" OR "Breathing" OR "Sucking Behavior" OR "Sucking Behaviors" OR "sucking") (Tópico)
**Google Scholar**	"Down Syndrome" AND "myofunctional therapy" AND "Deglutition" OR "Speech" OR "Respiration" OR "Breathing" OR "sucking" OR "Stomatognathic System"
**ProQuest**	noft("Down Syndrome" OR "Mongolism" OR "Trisomy G" OR "Down's Syndrome" OR "Downs Syndrome" OR "Trisomy 21") AND noft("myofunctional therapy" OR "myofunctional therapies" OR "orofacial myotherapy" OR "oral myotherapy" OR "orofacial myology" OR "oral exercise" OR "exercise therapy" OR "remedial exercise" OR "rehabilitation exercise" OR "habilitation" OR "therapeutics" OR "therapy" OR "Therapeutic" OR "Therapies" OR "Treatment" OR "Treatments" OR "intervention" OR "interventions") AND noft("Mastication" OR "chewing" OR "chewing ability" OR "chewing performance" OR "masticatory ability" OR "masticatory function" OR "masticatory disability" OR "masticatory dysfunction" OR "chewing function" OR "chewing discomfort" OR "chewing disorder" OR "chewing problems" OR "Sucking Behavior" OR "Sucking Behaviors" OR "sucking")
