# Supplementary material for: Speech-language-hearing interventions in orofacial functions in individuals with Down syndrome: a scoping review
Source: Codas. 2026 Mar 27;38(2):e20240337. doi: 10.1590/2317-1782/e20240337en (PMC13075832; doi:10.1590/2317-1782/e20240337en)
Supplement: Translation

# Translation: Intervenções fonoaudiológicas nas funções orofaciais na pessoa com trissomia do 21: uma revisão de escopo

**DOI:** 10.1590/2317-1782/e20240337pt

**Authors:** Paula Rayana Batista Correia, Maria Louize Justino Freire, Julyane Feitoza Coêlho, Manuela Leitão de Vasconcelos, Andréa Rodrigues Motta, Karinna Veríssimo Meira Taveira, Giorvan Anderson dos Santos Alves

**Affiliations:** 1 Programa Associado de Pós-graduação em Fonoaudiologia, Universidade Federal da Paraíba – UFPB - João Pessoa (PB), Brasil.; 2 Departamento de Fonoaudiologia, Universidade Federal de Minas Gerais – UFMG - Belo Horizonte (MG), Brasil.; 3 Departamento de Morfologia, Centro de Biociências, Universidade Federal do Rio Grande do Norte – UFRN - Natal (RN), Brasil.; 4 Núcleo de Estudos Avançados em Revisão Sistemática e Meta-análise – NARSM, Universidade Federal do Rio Grande do Norte – UFRN - Natal (RN), Brasil.; 5 Núcleo de Inteligência Artificial em Saúde – NIAS, Universidade Federal do Rio Grande do Norte – UFRN - Natal (RN), Brasil.

**Keywords:** Terapia Miofuncional, Terapia por Exercício, Reabilitação, Sistema Estomatognático, Sucção, Mastigação, Deglutição, Respiração, Fala, Revisão

## Abstract

**Objetivo:**

Mapear, na literatura, as sínteses de evidências sobre intervenções fonoaudiológicas nas funções orofaciais e seus efeitos na pessoa com trissomia do cromossomo 21 (T21).

**Estratégia de pesquisa:**

Foi realizada uma busca nas bases de dados EMBASE, LILACS, Pubmed/Medline, Scopus, Web of Science, Cochrane, ASHA e consulta na literatura cinzenta.

**Critérios de seleção:**

Foram incluídos estudos com pessoas com T21 que abordaram terapias relacionadas às funções orofaciais. Foram excluídos estudos com outra população, estudos em que a intervenção de interesse se caracterizou como terapia não fonoaudiológica e não direcionada para as funções orofaciais, além de estudos descritivos.

**Análise dos dados:**

Foram sintetizados e descritos de forma narrativa, ilustrados por meio de quadro e fluxograma.

**Resultados:**

Foram incluídos dez estudos. As publicações envolveram todas as funções, sendo a deglutição a mais recorrente delas. Os estudos foram publicados entre 2009 e 2024 e originaram-se de diversos países. Os participantes foram de ambos os sexos, com idade entre 2 e 25 anos. Parâmetros como número e frequência das sessões, bem como a duração do tratamento, foram variáveis. Os tipos de exercícios e intervenções, assim como os métodos avaliativos foram individualizados para cada estudo. Cinco estudos foram ensaios clínicos e realizaram avaliações quantitativas para verificação da efetividade das intervenções.

**Conclusão:**

Evidenciou-se, na literatura, que a intervenção fonoaudiológica nas funções orofaciais em indivíduos com T21 promove ganhos e modificações nos aspectos estruturais e funcionais.

## INTRODUÇÃO

Na população com Trissomia do cromossomo 21 (T21), observam-se alterações no crescimento maxilar e mandibular, como hipoplasia da maxila e prognatismo da mandíbula, além de más oclusões dentárias, palato estreito, língua alojada no assoalho da boca e/ou protrusa e hipotonia muscular^(1)^. Esses aspectos podem afetar a musculatura orofacial e prejudicar o funcionamento do sistema estomatognático (SE)^(1,2)^.

No que diz respeito aos aspectos respiratórios, é necessário um bom desempenho das estruturas orofaciais para que esta função ocorra de forma fisiológica, por via nasal. Há uma maior tendência para alterações respiratórias em pessoas com T21 como, a respiração oral e a apneia obstrutiva do sono (AOS), influenciando na qualidade do sono, na aprendizagem, memória, desempenho de tarefas, agitação e no surgimento de outras patologias^(3-5)^.

Na função de sucção, as configurações da língua, bem como as outras características orais, podem afetar esse sistema, proporcionando uma desorganização em seu padrão. Embora existam poucas evidências científicas, estudos relatam que bebês com T21 podem ter a eficácia da amamentação prejudicada, com atraso na obtenção de vácuo intraoral e duração adequada da sucção^(6,7)^.

Em pessoas com T21, a disfunção mastigatória é frequente durante a alimentação, podendo repercutir na boa nutrição e na qualidade de vida dos indivíduos, entretanto, é reduzido o número de pesquisas que investigam essa função^(8,9)^. A deglutição também pode ser afetada na T21, sendo realizada de forma mais complexa e com menos coordenação, devido a menor tonicidade e eficiência dos músculos. Nesse contexto, crianças com T21 podem sofrer taxas mais altas de seletividade alimentar, tosse durante a deglutição e função de deglutição anormal^(10,11)^.

Além disso, estudos indicam que pessoas com T21 podem apresentar tendência ao surgimento de transtorno de fala de origem musculoesquelética. Eles são os mais comuns, nos quais as alterações de fala originam-se em decorrência de alterações estruturais ósseas e musculares, repercutindo em distúrbios fonatórios, na articulação, ressonância e prosódia, influenciando na sua inteligibilidade de fala^(12-14)^.

Diante disso, a partir da terapia fonoaudiológica é possível oferecer exercícios e técnicas específicas, que auxiliem no melhor desempenho e funcionalidade das estruturas e das habilidades orais, favorecendo um melhor desenvolvimento do sistema estomatognático das pessoas com T21, permitindo uma melhor qualidade em amplos aspectos de vida^(15)^. Para isso, é necessário um respaldo científico que subsidie a utilização de estratégias que promovam segurança e eficiência na intervenção.

Considerando que são inexistentes estudos de revisão que abordem as intervenções fonoaudiológicas nas funções orofaciais em indivíduos com T21, esta pesquisa propôs realizar esse levantamento da literatura. Deste modo, será possível elucidar as principais evidências disponíveis na área e apontar as lacunas existentes, indicando o foco de interesse a ser desenvolvido em futuras pesquisas. Assim, o objetivo deste estudo foi mapear na literatura as sínteses de evidências sobre intervenções fonoaudiológicas nas funções orofaciais e seus efeitos na pessoa com T21.

## MÉTODO

Esta revisão de escopo foi realizada de acordo com as diretrizes do manual do Joanna Briggs Institute (JBI)^(16)^, portanto, tanto o protocolo quanto esta revisão foram elaborados utilizando o *checklist* do *Preferred Reporting Items for Systematic Reviews and Meta-Analyses – Extension for Scoping Reviews* (PRISMA-ScR)^(17)^. O protocolo para este estudo foi registrado na plataforma de registro *Open Science Framework* ^(18)^.

### Critérios de elegibilidade

O acrônimo PCC (Participantes, Conceito, Contexto) foi utilizado para avaliar os critérios de elegibilidade. Participantes: pessoas com trissomia do 21; Conceito: funções do Sistema Estomatognático (SE), como sucção, respiração, mastigação, deglutição e fala (incluindo erros residuais de fala; Contexto: terapia. Com base nesses critérios, foi formulada a seguinte pergunta de pesquisa: Quais as evidências científicas disponíveis na literatura sobre as intervenções fonoaudiológicas nas funções do sistema estomatognático em pessoas com trissomia do cromossomo 21?

Foram incluídos estudos com pessoas com trissomia do 21 e que abordaram terapias fonoaudiológicas relacionadas à sucção, respiração, mastigação, deglutição e fala (incluindo erros residuais de fala). Os tipos de estudos incluídos foram: ensaios clínicos, série de casos e estudos de casos.

Foram excluídos desta revisão estudos com outra população; estudos que apresentaram intervenção fonoaudiológica não direcionada para as funções orofaciais; estudos em que a intervenção de interesse se caracterizou como terapia não fonoaudiológica, ou seja, abordagens terapêuticas como a acupuntura, uso de medicamentos ou cirurgias; estudos descritivos (estudos de corte transversal, coorte e caso controle, secundários como resumos, revisões sistemáticas, narrativa, integrativa, meta-análise, carta ao editor, guidelines, guias de prática clínica e opiniões de especialistas). Não houve critérios de exclusão com base em etnia, sexo, idade, idioma ou ano de publicação do estudo.

### Fontes de informações e estratégias de busca

A estratégia de busca foi conduzida através de pesquisas eletrônicas em publicações científicas indexadas nas seguintes bases de dados: ASHA, Embase, Pubmed/Medline, Lilacs, Scopus, Cochrane e Web of Science. A literatura cinzenta também foi empregada como fonte de informações por meio do Google Scholar e Proquest. Além disso, foi realizada a busca manual nas referências dos estudos incluídos. Foram utilizados os operadores booleanos OR e AND para combinar os descritores (therapeutics, mastication, Deglutition, Speech, Respiration, Sucking Behavior, Down Syndrome) e seus correlatos. Esses termos foram selecionados considerando os Descritores em Ciências da Saúde - DeCS/MESH e EMTREE. Todas as buscas foram realizadas no dia 11 de agosto de 2023 e realizada uma atualização em 05 de fevereiro de 2025 (Apêndice A).

### Seleção

A seleção das fontes de evidência foi conduzida em três etapas, utilizando o site *Rayyan*® (Rayyan QCRI)^(19)^ como ferramenta para análise quanto à inclusão e exclusão de artigos, além da remoção de duplicados por meio do gerenciador de referência Endnote® (EndNote® X7 Thomson Reuters, Filadélfia, PA).

Foi conduzida uma etapa de calibração no site *Rayyan* com o objetivo de avaliar o nível de concordância entre os dois revisores independentes. Os critérios de elegibilidade foram aplicados para determinar tanto a inclusão quanto a exclusão dos estudos. Juntamente com a questão de revisão, foram analisados os títulos e resumos de 100 artigos selecionados a partir da amostragem da literatura.

Foi encontrada uma boa concordância para títulos e resumos, com a obtenção de valor > 0.8 no Coeficiente de Concordância de Kappa. Após isso, foi realizada a triagem com os demais estudos da primeira fase.

Na segunda fase, os textos completos foram lidos e os critérios de elegibilidade foram aplicados. Dois revisores realizaram a seleção de forma independente e quaisquer divergências foram resolvidas por um terceiro revisor. Cabe destacar que, em todas as etapas da coleta, um terceiro revisor independente, com maior experiência clínica, esteve presente para analisar e resolver as discordâncias, a fim de selecionar as pesquisas relevantes sobre o tema e orientar a prática com base no conhecimento científico.

### Extração e análise dos dados

Os dados foram extraídos de acordo com um protocolo específico, elaborado pelos autores. Foi realizada uma análise considerando não apenas os dados da publicação, mas também características metodológicas, como tamanho da amostra, mensuração de variáveis, método de análise. Também, foi considerada a descrição da população, dos processos de intervenções nas funções orofaciais (incluindo a quantidade de sessões, frequência e formato da intervenção, bem como a descrição dos exercícios) e da avaliação realizada (incluindo instrumentos utilizados e os resultados obtidos). Os parâmetros temporais foram considerados, assim como a identificação dos resultados (positivos, negativos ou neutros) e da eficácia do programa ou intervenção.

As análises e a apresentação dos dados foram sintetizadas e descritas de forma narrativa, sendo ilustradas por meio de quadro e fluxograma.

## RESULTADOS

### Seleção de fontes de evidências

Foram identificados 2028 artigos nas bases de dados e 433 estudos nos repositórios de literatura cinzenta, conforme ilustrado na Figura 1. Após a remoção das duplicatas, um total de 2429 citações foram selecionadas (fase 1). Com base na análise dos títulos e resumos, 2013 estudos foram excluídos, restando 49 artigos para recuperação e avaliação integral quanto à elegibilidade (fase 2). Desses, onze estudos não foram recuperados na íntegra. Após a avaliação dos textos completos, 28 estudos foram excluídos. Ao final do processo, dez estudos foram considerados elegíveis para esta revisão de escopo.

**Figura 1:** Fluxograma da seleção de fontes de evidências

### Características dos estudos

O Quadro 1 apresenta as configurações dos estudos incluídos, dividida em autores, ano, país, tipo do estudo, características dos participantes (amostra e idade), programas de treinamento (número de sessões, frequência de sessões, tipo de exercício), intervenções fonoaudiológicas, métodos avaliativos e resultados.

**Quadro 1:** Características dos estudos incluídos

**Autor, Ano, País**	**Tipo de estudo**	**Características dos participantes**		**Programa de treinamento**				**Avaliação dos resultados**	**Resultados**
		**Amostra**	**Idade**	**Número de sessões**	**Frequência e duração das sessões**	**Intervenções fonoaudiológicas (Funções orofaciais)**	**Descrição dos exercícios**		
Anichini et al.^(20)^, 2013. Itália	Estudos de casos	n= 7 (3 com SBW, 2 com SD e 2 com outro diagnóstico	4-14 anos	Participante SD1: NR Participante SD2: Contínuo	Participante SD1: NR Participante SD2: 3 vezes por semana Duração não informada	Macroglossia e Deglutição	Intervenção individualizada: Paciente SD1: Exercícios para corrigir a macroglossia (estímulo da praxia buco-facial) e para melhorar a linguagem Paciente SD2: Exercícios fonoaudiológicos	Avaliação fonoaudiológica: Registro dos parâmetros vocais, fonética e articulação, Comunicação gestual, Comunicação oral, Avaliação das habilidades gráfica, escrita, leitura, rítmica e níveis perceptivos, Motricidade global e fina, Esquema comportamental. - Grande atenção foi dada à execução dos movimentos buco-linguais, à observação dos músculos periorais, língua, lábios e mandíbula e à avaliação da respiração, deglutição e habilidades de mastigação. - Exame médico físico, laboratorial e exames instrumentais	Paciente SD1: Melhorias na concentração, comportamento, atenção e habilidades fonatórias, a protrusão da língua foi reduzida, o tônus muscular tornou-se mais forte Paciente SD2: Boas respostas na macroglossia, na deglutição atípica e linguagem
Putri et al.^(21)^, 2021. Indonésia	Ensaio clínico randomizado	n= 41 Grupo controle: 21 Grupo intervenção: 20	3-9 anos	21 dias/sessões	3 vezes ao dia, com 1 hora de sessão	Sucção	Exercício de sucção - sugar água pura usando canudos retos e circulares	Exame Clínico e Escala de função de teste enxaguatório bucal MRT-F Teste de enxaguamento bucal: a) Transferir 10 ml de água da tigela para a boca b) Fechar os lábios e segurar a água na boca c) Mover as duas bochechas simetricamente e mover as bochechas alternadamente e) Descarte da água na pia	As pontuações do primeiro dia do MRT-F no grupo de canudos circulares foram 1(30%), 2(35%), 3(20%) e 4(15%), enquanto no grupo de canudos retos foram 1(38,2%), 2(19%), 3(42,9%) e 4(0%), respectivamente. No final da terceira semana as pontuações foram 1(25%), 2(25%), 3(15%), 4(20%) e 5(15%), enquanto nos canudos retos foram 1(33%), 2(19%), 3(28,6%), 4(14,3%) e 5(4,8%). - Houve diferença significativa dos escores de MRT-F antes e depois dos exercícios. A capacidade de enxaguamento bucal em crianças com SD melhorou após a realização de exercícios de sucção de água em ambos os grupos. No entanto, não há diferenças significativas entre os exercícios com canudo reto e circular.
Saccomanno et al.^(22)^, 2018. Roma	Estudo Piloto de intervenção, não randomizado.	n= 10	9-18 anos	20 semanas	Cada objetivo: uma série de 5 exercícios. Em casa: repetir cada exercício pelo menos 3 vezes ao dia totalizando 30 min de TM por dia	1- Funcionalidade do nariz 2- Respiração nasal 3- Posição de língua 4- Selamento labial 5- Bucinador e músculos masseteres 6- Palato mole 7- deglutição	1º exercício: Higiene nasal: técnicas de lavagem das fossas nasais e aprender a assoar o nariz corretamente 2º exercício: Manter a boca fechada e inspirar e expirar, mantendo um abaixador de língua entre os lábios. 3º exercício: Estimular primeiro a porção anterior da língua, depois a porção lateral e por fim a posterior para adequar a posição de língua 4º exercício: Exercícios que restaurem o selamento labial 5º exercício: Exercícios de mastigação, estabilidade de mandíbula e exercício de bochecha inflada sustentada 6º Exercícios Gargarejo e pronúncia enérgica dos fonemas vocálicos e das vogais únicas 7º Exercício: Técnicas que repadronizem o movimento da língua para alcançar uma deglutição adequada	Avaliação Oromiofuncional, Avaliação cognitiva, Avaliação das habilidades de linguagem, tanto receptivas quanto expressivas, e um Exame de praxia língua-boca-face	Todos demonstraram boa habilidade em limpar e liberar as passagens nasais, assoando o nariz ou realizando lavagens nasais. Todos apresentaram capacidade de respiração nasal habitualmente. Quatro participantes conseguiram postura de língua em repouso no palato. Todos melhoraram no selamento labial durante a alimentação, e em repouso ainda eram inconsistentes. Todos tiveram melhora do tônus do masseter e da mastigação, assim como aumento na mobilidade do palato. Todos tiveram melhora na deglutição, reduziram sialorreia, rinorreia ou queilite angular
Von Lukowicz et al.^(23)^, 2019. Alemanha	Estudo experimental. Não Randomizado.	n= 42	2 – 11 anos	1 semana = 7 sessões	3 sessões diárias de 45 minutos por dia	Respiração – Apneia Obstrutiva do sono	Método Padovan: exercícios físicos que visam fortalecer o tônus muscular geral e melhorar a postura. Seguidos de exercícios orais, que visam ao controle do fluxo aéreo, atividade labial, movimentos da língua para aumentar sua força, ativação dos músculos bucinador e masseter e propriocepção por meio de exercícios de mastigação. Os exercícios orais visam fortalecer o complexo orofacial e melhorar a ingestão, a articulação e o desenvolvimento da face e dos maxilares	Exames noturnos de poligrafia (PG)	Não houve diferenças no MOAHI (índice de apneia/hipopneia obstrutiva, DI3(índice de dessaturação) ou SpO2nadir (menor valor de saturação de oxigênio) antes e depois da TM. Apenas o DI90 (índice de dessaturação) diminuiu. Além disso, o MOAHI mediano diminuiu mais em crianças sem TM prévia (de 6,1 para 1,5 vs de 2,7 para 2,2)
Cleland et al.^(24)^, 2009. Escócia	Ensaio clínico randomizado	n= 6	10 – 18 anos	24 sessões de terapia individualizada	2 vezes por semana durante um período de 12 semanas 1 hora na clínica. Em casa: 10 a 15 minutos, 5 dias por semana	Fala	Início com fonema alvo em CV ou VC progredindo para CV e VC com vogais diferentes, até palavras (complexidade de construção) e, em seguida, frases e conversação no discurso. Inicialmente os participantes foram incentivados a copiar o padrão alvo usando o EPG para feedback visual, com progressos para produções sem feedback visual Programa individualizado	Avaliação de linguagem: (BPVS-II, Dunn et al.^(25)^) CELF-P, Wiig et al.^(26)^). Habilidade Cognitiva: (Escala Wechsler de Inteligência Pré-escolar e Primária e (WPPSI-IIIUK, Wechsler^(27)^. Função oromotora: (Avaliação clínica de Robbins e Klee^(28)^) PRÉ E PÓS: - Medidas EPG. - Fonologia (DEAP, Dodd et al.^(29)^)	Houve um aumento significativo no PCC de 63,50% para 73,67% (t(5) = 3,634, p = 0,015), com um aumento médio, por criança, de 10,17%. Houve um rápido aumento, mas não significativo, de 63,5% para 66,06% no número de palavras corretamente identificadas pelos ouvintes pós-intervenção (t(5) = 0,616, p = 0,565) no CSIM. Como grupo, as mudanças positivas são evidentes em cinco destes seis participantes, com o participante 2 não mostrando nenhuma alteração nos padrões do EPG, mas uma mudança para um fluxo de ar mais apropriado. Perceptivamente, todas as crianças, exceto a participante 2, apresentam um aumento no número de produções perceptivamente corretas. No conjunto este aumento é significativo (t(5) = 3,286; Todos os participantes mostraram melhorias na porcentagem de consoantes corretas e a maioria dos participantes produziu padrões EPG visivelmente diferentes após 24 sessões de terapia de feedback visual EPG.
Alves Pinheiro et al.^(2)^, 2018. Brasil	Estudo de intervenção não randomizado	n= 16	9-25 anos	8 sessões	Semanal Duração não informada	Mastigação	Treino mastigatório (com biscoito Bono), sempre com comandos para induzir os voluntários a mastigarem de forma bilateral e alternada e, quando possível, com selamento labial. Eletroestimulação	Avaliação Miofuncional Orofacial através do AMIOFE	Mudança significativa em relação à alteração postural dos lábios, mantendo-se lábios selados Mudança significativa em relação ao aspecto das bochechas, diminuindo a flacidez Diferença pré e pós na lateralização de língua, tanto para o lado direito quanto para o esquerdo Melhoria da respiração Melhoria no comportamento dos lábios durante a deglutição. Na mastigação, a mordida e a trituração foram aspectos que destacaram diferença significativa, quando comparadas pré e pós-intervenção. A mordida teve a busca para ser realizada pelos incisivos e a trituração buscou o padrão de normalidade bilateral alternado
Wood et al.^(30)^, 2018. Escócia	Ensaio clínico randomizado	n= 27 Terapia grupo 1 = EPG diretamente VBF Terapia grupo 2= Terapia convencional informada por avaliação EPG pré-terapia Terapia grupo 3= Grupo de controle, tratamento usual.	8-18 anos	Durante 12 semanas 24 sessões	Duas vezes por semana, com sessões de 1 hora	Fala	Terapia de fala associada ao EPG: A terapia seguiu uma hierarquia articulatória básica começando com o fonema alvo em CV ou estrutura VC usando uma vogal facilitadora e progredindo para outras vogais e estruturas CVC e continuando para estruturas de palavras mais complexas. Onde o progresso indicou tal possibilidade, o SLT passou para o nível de frase e sentença	Capacidade cognitiva: Escala (WPPSI-IIIUK; Wechsler^(27)^ Vocabulário receptivo:(BPVS-II; Dunn et al.^(25)^) Linguagem receptiva e expressiva: (CELFP-UK, Wiig et al.^(26)^): Função oromotora: Avaliação clínica do desenvolvimento motor orofaríngeo^(28)^ Produção de fala: Subteste de Fonologia, Avaliação da Articulação e Fonologia (DEAP; Dodd et al.^(29)^): Inteligibilidade: Medida de Inteligibilidade da Fala Infantil (CSIM; Wilcox e Morris^(31)^)	De acordo com os escores de diferença do DEAP PCC pré e pós-terapia não houve diferença estatisticamente significativa entre os grupos de intervenção neste momento: ÿ2 (2) = 1,830, p = 0,00. 401
Homer e Carbajal^(32)^, 2015. Estados Unidos	Estudo de caso	N=1	5 anos	NR	Diariamente Duração não informada	Deglutição	Plano de deglutição e alimentação que aborda posicionamento, equipamentos, estado de alimentação, dieta, preparo dos alimentos e técnicas e precauções do plano de alimentação. Dessensibilização através da estimulação motora oral usando uma escova Nuk antes das refeições e incorporando exercícios para melhorar a lateralização da língua, o fechamento dos lábios e as habilidades motoras orais gerais para comer. Técnicas para reduzir a protrusão da língua. Tentativas de copos diferentes e diminuir o uso de canudo. Aumentar gradualmente a variedade de alimentos, as texturas deles e a quantidade de alimentos e líquidos consumidos durante o dia escolar, adicionando um ou dois novos alimentos de cada vez. Técnicas para promover a alimentação independente, incluindo encorajar a alimentação com os dedos e com a colher para boca colocando-os em seus molares e dando instruções verbais para ele mastigar. Técnicas para autoalimentação	NR	Ajudou a aumentar sua tolerância a texturas aumentadas e a estimular a mastigação.
Junqueira et al.^(33)^, 2023. Brasil	Estudo de caso	N=1	3 anos e 2 meses	6 meses	1 vez por semana Duração não informada	Mastigação	Estímulos vestibulares (balanço), táteis e proprioceptivos (atividades de força, vibração e massagens Estimulação tátil-térmico-gustativa, com uso de água saborizada com fruta cítrica e mordedores texturizados. Os alimentos eram ofertados ao longo da sessão, estimulando a colocação do alimento na região dos molares, favorecendo a lateralização da língua e, consequentemente, a mastigação. Eram ofertadas diversas formas de apresentação do mesmo alimento: inteiro, cortado, esfarelado, com dedo, com garfo, com colher texturizada etc., promovendo variações no padrão sensório-mo-tor-oral.	Avaliação fonoaudiológica foi realizada presencialmente com o uso de alimentos pelos quais a criança manifestava interesse e com auxílio de utensílios habituais (colher e mordedores).	Houve ampliação do cardápio para a ingestão de todos os tipos de alimento, a melhora da percepção e da habilidade motora intraoral, a aceitação de colheres e garfos de diferentes tamanhos e modos de apresentação do alimento (inteiros, cortados e esfarelados - ofe-recidos com talheres ou com as mãos), autonomia e prazer nas refeições.
Franceschetti et al.^(34)^, 2024. Itália	Estudo Piloto de intervenção, não randomizado.	N= 20	1-18 anos	15 sessões	3 sessões por dia, 5 dias consecutivos Duração não informada	Mastigação	Programa de Terapia de alimentação intensiva global (GIFT)	Triagem Pediátrica – Avaliação Prioritária de Disfagia (PS–PED) Escala de Desempenho de Mastigação de Karaduman (KCPS) Iniciativa Internacional de Padronização da Dieta para Disfagia (IDDSI)	Melhorias estatisticamente significativas no desempenho da mastigação foram observadas, conforme medido pelo KCPS (*p*< 0,01), bem como na aceitação e modificação da textura, conforme medido pelo IDDSI pós-intervenção (*p*< 0,01). Para o KCPS e o IDDSI, um grande tamanho de efeito foi encontrado (valor do Kendall's W > 0,8).

Legenda: SBW: Síndrome de Beckwith-Wiedemann; SD: Síndrome de Down; SD1: Paciente Síndrome de Down número 1; SD2: Paciente Síndrome de Down número 2; NR: Não reportado; EPG: Eletropalatografia; VBF: Biofeedback visual; PCC: porcentagem de consoantes corretas; MOAHI: Índice misto de apneia/hipopneia obstrutiva; DI90: Índice de dessaturação; CV: Consoante Vogal; VC: Vogal Consoante; CVC: Consoante Vogal Consoante

Os estudos incluídos foram publicados entre 2009 e 2024 em diferentes periódicos. A pesquisa foi conduzida em diversos países, como Brasil^(2,33)^, Itália^(20,22,34)^, Indonésia^(21)^, Alemanha^(23)^, Escócia^(24,30)^ e Estados Unidos^(32)^.

Todos os participantes tinham diagnóstico de T21, abrangendo ambos os sexos, com idades variando entre 2 e 25 anos. Um estudo abordou a deglutição e outros aspectos estruturais do SE^(20)^, outro focou exclusivamente na sucção^(21)^, um abrangeu respiração, mastigação e deglutição^(22)^, enquanto outro se concentrou na respiração^(23)^. Dois analisaram a fala^(24,30)^, três a mastigação^(2,33,34)^ e um abordou unicamente a deglutição^(32)^.

O número de sessões variou significativamente, indo de sete até tratamento contínuo. Um estudo envolveu apenas uma sessão terapêutica que foi complementada por capacitação dos familiares para realizar a intervenção em casa^(21)^. A frequência do tratamento também foi bastante distinta entre os estudos. A duração dos tratamentos variou de 1 a 12 semanas, sendo que alguns estudos não descreveram essas informações detalhadamente^(20,32)^. Os tipos de exercícios e intervenções também foram específicos de cada estudo, com alguns sendo individualizados e outros seguindo o mesmo padrão para todos os participantes. Nos métodos de avaliação, foram utilizados protocolos e técnicas específicas para cada área de interesse.

Dentre os estudos, três foram ensaios clínicos randomizados^(21,24,30)^, quatro ensaios clínicos não randomizados^(2,22,23,34)^ e três estudos de casos^(20,32,33)^. Em cinco dos estudos incluídos, foram realizadas avaliações qualitativas para verificar a efetividade das intervenções^(20,22,23,32,33)^, enquanto cinco estudos realizaram análises quantitativas^(2,21,24,30,34)^.

Alguns estudos sugeriram aumentar o tamanho da amostra, considerar a capacidade cognitiva, a gravidade das alterações estruturais e funcionais, o acesso a outros tratamentos, o tempo de intervenção e o protocolo de intervenção para alcançar a estabilidade dos resultados ao longo do tempo^(21,23,30,34)^.

### Resultados individuais dos estudos

#### Sucção

Foi localizado apenas um estudo interventivo na função de sucção em pessoas com T21^(21)^. Nesse estudo, foram utilizados exercícios de sugar água pura, por meio de canudos retos e circulares. Os dados foram avaliados quantitativamente, demonstrando que a capacidade de enxaguamento bucal melhorou após a realização dos exercícios, em ambos os grupos. No entanto, não houve diferenças significativas entre os exercícios com canudo reto e circular, sugerindo a necessidade de maior investigação, com modificações metodológicas^(21)^.

#### Respiração

Foi identificado um estudo^(23)^ relacionado à Apneia Obstrutiva do Sono (AOS), que envolveu intervenções baseadas em exercícios do Método Padovan. Após uma análise quantitativa, concluiu-se que o treinamento miofuncional intenso, realizado durante uma semana e avaliado em crianças com T21, teve apenas um efeito marginal na AOS. O estudo sugere que um período de acompanhamento mais longo ou o aumento da duração da intervenção poderiam produzir efeitos mais significativos^(23)^.

#### Mastigação

Três estudos foram encontrados sobre a mastigação, no qual a intervenção de um deles consistiu em treino mastigatório associado à aplicação da eletroestimulação^(2)^. Os resultados, analisados estatisticamente, mostraram que a eletroestimulação combinada com o treino mastigatório teve efeitos positivos nos músculos masseteres, resultando em ganhos funcionais na mastigação, respiração e deglutição em pessoas com T21^(2)^.

O segundo^(33)^ apresentou estímulos vestibulares e táteis, proprioceptivos, atividades de força, vibração, massagem e treino mastigatório. Os resultados, analisados qualitativamente, demonstraram que houve melhora na habilidade motora intraoral, autonomia e prazer nas refeições^(33)^.

Já no terceiro^(23)^, a intervenção foi baseada em um programa de terapia de alimentação intensiva (GIFT) com técnicas que proporcionaram resultados estatisticamente significativos no desempenho da mastigação^(23)^.

#### Deglutição

Em um dos estudos sobre deglutição^(20)^, as intervenções foram individualizadas para cada paciente, com a aplicação de exercícios para correção da macroglossia, estímulos de praxia orofacial, exercícios voltados para linguagem e exercícios fonoaudiológicos. Os resultados, expressos qualitativamente, indicaram que todas as crianças do estudo apresentaram boa resposta e adesão, com melhorias notáveis, demonstrando a importância de um tratamento precoce, constante e intensivo^(20)^.

Outro estudo, focado na deglutição^(32)^, apresentou um plano de intervenção para deglutição e alimentação. Os resultados, também foram expressos qualitativamente, concluindo-se que a intervenção contribuiu para aumentar a tolerância a texturas alimentares mais densas e para estimular a mastigação^(32)^.

#### Fala

Em um dos estudos sobre a função de fala^(24)^, as intervenções foram individualizadas, seguindo um padrão hierárquico, começando com fonema - alvo e progredindo para variações, palavras, frases e conversação no discurso. Também foi utilizada a eletropalatografia (EPG) para *feedback* visual, com progressos até produções sem esse feedback. Os resultados, avaliados quantitativamente, mostraram diferenças qualitativas e quantificáveis nos padrões EPG e melhorias na porcentagem de consoantes corretas, indicando que a EPG é uma abordagem promissora para identificar e melhorar os padrões articulatórios em pessoas com T21^(24)^.

De forma concordante, em outro estudo referente à fala^(30)^, no qual os participantes receberam terapia com EPG, após a análise estatística, verificou-se que a intervenção foi eficaz na melhora da produção da fala em muitos participantes. Entretanto, os autores ressaltaram a necessidade de investigação mais aprofundada com amostra maior^(30)^.

#### Funções estomatognáticas e aspectos estruturais

Um estudo abordou um protocolo de intervenção para T21, com exercícios relacionados aos seguintes aspectos: funcionalidade do nariz; respiração nasal; posição de língua; selamento labial; fortalecimento e mobilidade de músculos bucinadores e masseteres; palato mole; e deglutição. Os resultados foram descritos de forma qualitativa, indicando que houve melhoras em vários aspectos trabalhados, como a restauração adequada da cinética fisiológica orofacial e das funções orais como a deglutição, a respiração e a articulação^(22)^.

## DISCUSSÃO

As intervenções fonoaudiológicas voltadas às funções orofaciais em indivíduos com T21 demonstraram potencial para promover melhorias clínicas relevantes em aspectos como sucção, respiração, mastigação, deglutição e fala. Esses resultados reforçam a importância da atuação fonoaudiológica na promoção da funcionalidade do sistema estomatognático nessa população.

Os estudos incluídos foram conduzidos entre 2009 e 2024^(2,20-24,30,32-34)^, sendo apenas três deles publicados nos últimos cinco anos^(21,33,34)^, deste modo, evidencia-se a escassez de pesquisas na área e ressalta-se a necessidade iminente de expansão.

Os participantes dos estudos foram principalmente crianças e adolescentes com T21^(20-24,30,33,34)^, enquanto apenas um incluiu adultos^(2)^ com T21. Tais achados demonstram uma maior tendência de estudos voltados para os períodos iniciais da vida, podendo estar relacionada à ênfase na estimulação fonoaudiológica precoce, que ocorre nos primeiros anos de vida^(15,35)^.

Observou-se que a maioria dos estudos apresentou um número reduzido de participantes com T21, variando de 1 a 20 indivíduos^(2,20-22,24,30,32-34)^, sendo que, apenas um apresentou uma amostra maior, composta por 42 indivíduos^(23)^. Esses aspectos podem estar relacionados às dificuldades no recrutamento de participantes e na adesão ao processo terapêutico, desafios comuns em pesquisas científicas com essa população.

Outras considerações metodológicas relevantes, observadas em grande parte dos estudos, incluem a ausência de grupo controle^(2,20,22,23,32,34)^ e a escolha pela utilização de análises qualitativas para avaliar os efeitos das intervenções^(20,22,23,32,33)^. Esses fatores dificultam o entendimento de quais aspectos diferenciais devem ser implementados nas intervenções voltadas para essa população, pela falta de parâmetros comparativos obtidos junto a indivíduos sem a T21 submetidos à mesma intervenção.

No que se refere à intervenção sobre a sucção em T21^(21)^, os exercícios incluíram a sucção de água por meio do canudo, com frequência de três vezes ao dia, por uma hora. Entretanto, os próprios autores sugeriram mudanças metodológicas para obtenção de resultados mais consistentes^(23)^. A dificuldade na sucção em bebês com T21 é um fator relevante, uma vez que pode comprometer a amamentação devido ao atraso na obtenção de vácuo intraoral e na duração da sucção^(6,7,36)^.

Na respiração, os resultados com o Método Padovan não foram satisfatórios na AOS, possivelmente devido à curta duração do acompanhamento e ao tamanho reduzido da amostra^(23)^. Esse método consiste em uma sequência de exercícios motores, respiratórios e estímulos reflexo-vegetativos orais, com impacto potencial na função respiratória^(37)^.

Na mastigação, apenas um estudo utilizou o treino mastigatório associado à eletroestimulação, demonstrando efeitos positivos na população com T21. Além disso, a intervenção específica na musculatura mastigatória promoveu melhorias em outras funções estomatognáticas^(2)^. Enquanto os outros dois utilizaram terapia convencional da área, sem o auxílio de recursos tecnológicos, também apresentando bons resultados tanto estruturais quanto funcionais^(33,34)^.

Quanto à deglutição, os achados foram apresentados qualitativamente, revelando melhorias no posicionamento e tonicidade das estruturas orofaciais, além do aumento na tolerância a texturas mais firmes^(20,32)^. Essas informações reforçam a necessidade de estudos controlados para avaliar a eficácia dessas intervenções em longo prazo.

Nos aspectos relativos à fala, um dos estudos mostrou melhorias na porcentagem de consoantes corretas e padrões diferenciados de eletropalatograma após intervenção^(24)^. Outro estudo comparou a terapia convencional com a terapia mediada por feedback visual via eletropalatografia, sem diferenças estatisticamente significantes entre os grupos, embora tenha havido ganho na precisão da produção^(30)^.

Um dos estudos envolveu intervenção em mais de uma função, alcançando melhorias funcionais na deglutição, respiração e fala^(22)^. É provável que a estimulação tenha proporcionado mudança nos aspectos neuromusculares, potencializando não só uma função do SE.

Os achados analisados indicam que a estimulação fonoaudiológica voltada para as funções orofaciais na T21 promove efeitos benéficos, incluindo melhorias em funções não diretamente trabalhadas^(2,21,32)^. Esse resultado corrobora a proposta da Terapia Miofuncional Orofacial, que busca recuperar a funcionalidade do sistema estomatognático por meio de técnicas que facilitam a ação neuromuscular^(38)^.

As abordagens convencionais foram predominantes entre os estudos incluídos^(20-23,32-34)^_,_ enquanto algumas pesquisas utilizaram recursos tecnológicos para potencializar os efeitos da intervenção^(2,24,30)^. A escassez de estudos na área evidencia a necessidade de aprofundamento das investigações para a obtenção de maior respaldo científico.

Entre as limitações identificadas, destacam-se a dificuldade de acesso a textos completos e o pouco detalhamento de alguns métodos de intervenção, dificultando a extração de dados. Além disso, a ausência de padronização nos protocolos de avaliação e intervenção compromete a comparabilidade dos resultados. O tamanho reduzido das amostras também limita a realização de análises quantitativas mais precisas, dificultando a obtenção de evidências mais robustas.

Por fim, a maioria dos estudos não utilizou ensaios clínicos randomizados controlados, considerados padrão-ouro para investigações em saúde^(39)^. Esse fator reforça a necessidade de pesquisas metodologicamente mais rigorosas para aprimorar as evidências na área.

A quantidade limitada de estudos disponíveis destaca a urgência de ampliação das investigações, com ênfase em amostras maiores, grupos controle, análises quantitativas robustas e acompanhamento longitudinal. Estudos futuros devem explorar as diversas funções orofaciais, especialmente a deglutição, que foi investigada em apenas dois estudos de caso. Além disso, recomenda-se a padronização de abordagens terapêuticas específicas para cada função, considerando diferentes faixas etárias e propondo estratégias baseadas nas melhores evidências disponíveis em Motricidade Orofacial.

## CONCLUSÃO

Na sucção, os resultados demonstraram evidências de melhorias na função, com base em um teste enxaguatório bucal. No que se refere à função de respiração, em indivíduos com apneia obstrutiva do sono, a intervenção resultou em um efeito apenas marginal na AOS. Além disso, intervenções direcionadas às funções de respiração e deglutição promoveram adequações não só nessas funções orais, mas também na articulação da fala.

Os achados relativos à mastigação demonstraram que a eletroestimulação associada ao treino mastigatório proporcionou ganhos funcionais nas funções de mastigação, respiração e deglutição; assim como a terapia convencional resultou em melhora no desempenho da mastigação, na habilidade motora intraoral, promovendo autonomia e prazer nas refeições. Estudos focados na deglutição demonstraram melhorias na função, aumento da tolerância a texturas mais densas e estímulo à mastigação. Nos estudos envolvendo a fala, foram identificadas modificações nos padrões eletropalatográficos e melhorias na produção da fala após a intervenção.

O presente estudo traz importantes contribuições no contexto clínico, uma vez que aponta as evidências disponíveis na intervenção direcionada às funções orofaciais, norteando os profissionais na elaboração de estratégias terapêuticas mais eficazes, principalmente os que atuam com intervenção direcionada às pessoas com a trissomia do 21, por meio de técnicas que potencializem a intervenção.

## Apêndice A Estratégias de busca

**Table:**

**Base de dados**	**Busca** (11 de agosto de 2023 e atualização em 05 de fevereiro de 2025)
**ASHA**	("Down Syndrome" OR "Mongolism" OR "Trisomy G" OR "Down's Syndrome" OR "Downs Syndrome" OR "Trisomy 21") AND ("myofunctional therapy" OR "myofunctional therapies" OR "orofacial myotherapy" OR "oral myotherapy" OR "orofacial myology" OR "oral exercise" OR "exercise therapy" OR "remedial exercise" OR "rehabilitation exercise" OR "habilitation" OR "therapeutics" OR "therapy" OR "Therapeutic" OR "Therapies" OR "Treatment" OR "Treatments" OR "intervention" OR "interventions") AND ("Mastication" OR "chewing" OR "chewing ability" OR "chewing performance" OR "masticatory ability" OR "masticatory function" OR "masticatory disability" OR "masticatory dysfunction" OR "chewing function" OR "chewing discomfort" OR "chewing disorder" OR "chewing problems" OR "Sucking Behavior" OR "Sucking Behaviors" OR "sucking")
**Cochrane**	("Down Syndrome" OR "Mongolism" OR "Trisomy G" OR "Down's Syndrome" OR "Downs Syndrome" OR "Trisomy 21") in Title Abstract Keyword AND ("myofunctional therapy" OR "myofunctional therapies" OR "orofacial myotherapy" OR "oral myotherapy" OR "orofacial myology" OR "oral exercise" OR "exercise therapy" OR "remedial exercise" OR "rehabilitation exercise" OR "habilitation" OR "therapeutics" OR "therapy" OR "Therapeutic" OR "Therapies" OR "Treatment" OR "Treatments" OR "intervention" OR "interventions") in Title Abstract Keyword AND ("Mastication" OR "chewing" OR "chewing ability" OR "chewing performance" OR "masticatory ability" OR "masticatory function" OR "masticatory disability" OR "masticatory dysfunction" OR "chewing function" OR "chewing discomfort" OR "chewing disorder" OR "chewing problems" OR "Sucking Behavior" OR "Sucking Behaviors" OR "sucking") in Title Abstract Keyword
**Embase**	#1. 'down syndrome'/exp OR 'down syndrome' OR 'mongolism'/exp OR 'mongolism' OR 'trisomy g'/exp OR 'trisomy g' OR 'downs syndrome'/exp OR 'downs syndrome' OR 'trisomy 21'/exp OR 'trisomy 21'
	#2. 'myofunctional therapy' OR 'myofunctional therapies' OR 'orofacial myotherapy' OR 'oral myotherapy' OR 'orofacial myology' OR 'oral exercise' OR 'promotion program' OR 'long-term care prevention programs' OR 'resistance training' OR 'strength training' OR 'exercise program' OR 'exercise therapy' OR 'remedial exercise'
	#3. 'stomatognathic system' OR 'masticatory system' OR 'mastication' OR 'chewing' OR 'deglutition' OR 'deglutitions' OR 'swallowing' OR 'swallowings' OR 'speech' OR 'respiration' OR 'breathing' OR 'sucking behavior' OR 'sucking behaviors' OR 'sucking'
	#4. #1 AND #2 AND #3
**LILACS**	("Down Syndrome" OR "Mongolism" OR "Trisomy G" OR "Down's Syndrome" OR "Downs Syndrome" OR "Trisomy 21") AND ("Myofunctional Therapy" OR "myofunctional therapies" OR "orofacial myotherapy" OR "oral myotherapy" OR "orofacial myology" OR "oral exercise" OR "exercise therapy" OR "remedial exercise" OR "rehabilitation exercise" OR "rehabilitation" OR "habilitation" OR "terapia miofuncional" OR "treinamento de força" OR "terapia por exercício" OR "reabilitação" OR "habilitação" OR "terapia miofuncional" OR "entrenamiento de fuerza" OR "terapia por ejercicio" OR "rehabilitación" OR "habilitacion") AND ("Stomatognathic System" OR "Masticatory System" OR "Mastication" OR "Chewing" OR "Deglutition" OR "Deglutitions" OR "Swallowing" OR "Speech" OR "Respiration" OR "Breathing" OR "Sucking Behavior" OR "Sucking Behaviors" OR "sucking" OR "sistema estomatognático" OR "sistema mastigatório" OR "mastigação" OR "deglutição" OR "fala" OR "respiração" OR "sistema estomatognático" OR "sistema masticatorio" OR "masticación" OR "deglución" OR "habla" OR "respiración" OR "Comportamento de Sucção" OR "Conducta en la Lactancia")
**PubMed/Medline**	#1. ("Down Syndrome"[Mesh] OR "Down Syndrome" OR "Mongolism" OR "Trisomy G" OR "Down's Syndrome" OR "Downs Syndrome" OR "Trisomy 21")
	#2. ("Myofunctional Therapy"[MeSH] OR "myofunctional therapy" OR "myofunctional therapies" OR "orofacial myotherapy" OR "oral myotherapy" OR "orofacial myology" OR "oral exercise" OR "exercise therapy"[MeSH] OR "remedial exercise" OR "rehabilitation exercise" OR "rehabilitation"[MeSH] OR "habilitation" OR "therapeutics"[MesH] OR "therapeutics" OR "therapy" OR "Therapeutic" OR "Therapies" OR "Treatment" OR "Treatments" OR "intervention" OR "interventions")
	#3. ("Stomatognathic System"[MeSH] OR "Masticatory System" OR "Mastication"[MesH] OR "mastication" OR "Chewing" OR "Deglutition"[Mesh] OR "Deglutitions" OR "Swallowing" OR "Swallowings" OR "Speech"[Mesh] OR "Respiration"[Mesh] OR "Breathing" OR "Sucking Behavior"[Mesh] OR "Sucking Behavior" OR "Sucking Behaviors" OR "sucking")
	#4. #1 AND #2 AND #3
**Scopus**	("Down Syndrome" OR "Mongolism" OR "Trisomy G" OR "Down's Syndrome" OR "Downs Syndrome" OR "Trisomy 21") AND TITLE-ABS-KEY ("myofunctional therapy" OR "myofunctional therapies" OR "orofacial myotherapy" OR "oral myotherapy" OR "orofacial myology" OR "oral exercise" OR "exercise therapy" OR "remedial exercise" OR "rehabilitation exercise" OR "habilitation" OR "therapeutics" OR "therapy" OR "Therapeutic" OR "Therapies" OR "Treatment" OR "Treatments" OR "intervention" OR "interventions") AND TITLE-ABS-KEY ( "Stomatognathic System" OR "Masticatory System" OR "mastication" OR "Chewing" OR "Deglutition" OR "Deglutitions" OR "Swallowing" OR "Swallowings" OR "Speech" OR "Respiration" OR "Breathing" OR "Sucking Behavior" OR "Sucking Behaviors" OR "sucking")
**Web of Science**	("Down Syndrome" OR "Mongolism" OR "Trisomy G" OR "Down's Syndrome" OR "Downs Syndrome" OR "Trisomy 21") (Tópico) AND ("myofunctional therapy" OR "myofunctional therapies" OR "orofacial myotherapy" OR "oral myotherapy" OR "orofacial myology" OR "oral exercise" OR "exercise therapy" OR "remedial exercise" OR "rehabilitation exercise" OR "habilitation" OR "therapeutics" OR "therapy" OR "Therapeutic" OR "Therapies" OR "Treatment" OR "Treatments" OR "intervention" OR "interventions") (Tópico) AND ("Stomatognathic System" OR "Masticatory System" OR "mastication" OR "Chewing" OR "Deglutition" OR "deglutition" OR "Swallowing" OR "swallowing" OR "Speech" OR "Respiration" OR "Breathing" OR "Sucking Behavior" OR "Sucking Behaviors" OR "sucking") (Tópico)
**Google Scholar**	"Down Syndrome" AND "myofunctional therapy" AND "Deglutition" OR "Speech" OR "Respiration" OR "Breathing" OR "sucking" OR "Stomatognathic System"
**ProQuest**	noft("Down Syndrome" OR "Mongolism" OR "Trisomy G" OR "Down's Syndrome" OR "Downs Syndrome" OR "Trisomy 21") AND noft("myofunctional therapy" OR "myofunctional therapies" OR "orofacial myotherapy" OR "oral myotherapy" OR "orofacial myology" OR "oral exercise" OR "exercise therapy" OR "remedial exercise" OR "rehabilitation exercise" OR "habilitation" OR "therapeutics" OR "therapy" OR "Therapeutic" OR "Therapies" OR "Treatment" OR "Treatments" OR "intervention" OR "interventions") AND noft("Mastication" OR "chewing" OR "chewing ability" OR "chewing performance" OR "masticatory ability" OR "masticatory function" OR "masticatory disability" OR "masticatory dysfunction" OR "chewing function" OR "chewing discomfort" OR "chewing disorder" OR "chewing problems" OR "Sucking Behavior" OR "Sucking Behaviors" OR "sucking")
